# Vulnerability of primary neurons derived from Tg2576 Alzheimer mice to oxygen and glucose deprivation: role of intraneuronal amyloid-β accumulation and astrocytes

**DOI:** 10.1242/dmm.028001

**Published:** 2017-05-01

**Authors:** Vito Antonio Baldassarro, Alessandra Marchesini, Luciana Giardino, Laura Calzà

**Affiliations:** 1 Interdepartmental Centre for Industrial Research in Health Science and Technologies (ICIR - HST), University of Bologna, 40064 Ozzano Emilia, Bologna, Italy; 2 Department of Pharmacy and Biotechnology (FaBit), University of Bologna, 40127 Bologna, Italy; 3 Fondazione IRET, 40064 Ozzano Emilia, Bologna, Italy; 4 Department of Medical Veterinary Sciences (DIMEVET), University of Bologna, 40064 Ozzano Emilia, Bologna, Italy

**Keywords:** Alzheimer's disease, Primary neurons, Intraneuronal amyloid, Oxygen glucose deprivation, Glutamate, Neurovascular coupling

## Abstract

Microvascular dysfunction is considered an integral part of Alzheimer disease (AD) pathogenesis, but the possible relationship between amyloid pathology, microvascular dysfunction and cell death is still unclear. In order to investigate the influence of intraneuronal amyloid-β (Aβ) accumulation on vulnerability to hypoxia, we isolated primary cortical neurons from Tg2576 (carrying the amyloid precursor protein APPSwe mutation) and wild-type fetal mice. We first demonstrated that neurons isolated from Tg2576 newborn mice show an increase in VEGFa mRNA expression and a decrease in the expression of the two VEGF receptors, Flt1 and Kdr, compared with wild-type cells. Moreover, APPSwe primary neurons displayed higher spontaneous and glutamate-induced cell death. We then deprived the cultures of oxygen and glucose (OGD) as an *in vitro* model of hypoxia. After OGD, APPSwe neurons display higher levels of cell death in terms of percentage of pyknotic/fragmented nuclei and mitochondrial depolarization, accompanied by an increase in the intraneuronal Aβ content. To explore the influence of intraneuronal Aβ peptide accumulation, we used the γ-secretase inhibitor LY450139, which showed that the reduction of the intracellular amyloid fully protects APPSwe neurons from OGD-induced degeneration. Conditioned medium from OGD-exposed APPSwe or wild-type astrocytes protected APPswe neurons but not wild-type neurons, during OGD. In conclusion, the presence of the mutated human APP gene, leading to the intracellular accumulation of APP and Aβ fragments, worsens OGD toxicity. Protection of APPSwe neurons can be obtained either using a γ-secretase inhibitor or astrocyte conditioned medium.

## Abstract (precis)

**Summary:** *In vitro* systems derived from AD mice can be used to investigate the vulnerability of AD neurons to different neurotoxic challenges, including oxygen glucose deprivation.

## INTRODUCTION

Brain function is strictly dependent on an appropriate blood support and tissue perfusion, to ensure nutrient and oxygen delivery and to remove metabolic waste products (Zlokovic, 2011). The fine regulation of the blood support to the neurons is performed by the cerebrovascular unit (CVU) which provides the functional coupling between energy demand and vasodilation (Nelson et al., 2016). This histological structure includes neurons, vascular cells (endothelial cells, pericytes and vascular smooth muscle cells), glial cells (astrocytes, microglia, and oligodendrocytes) and extracellular matrix protein, which plays a part in blood–brain barrier (BBB) regulation. Notably, the paracrine mechanism, including vascular endothelial growth factor (VEGF) and related receptors, participates in the cross-talk among different cell types in both normal and ischemic conditions (Redzic et al., 2015). To highlight the importance of the glial component, namely astrocytes, the CVU is also termed ‘gliovascular unit’. In the CVU, astrocytes define functional domains and contact the microvessels with endfeet plastered on the vessel wall (Nedergaard et al., 2003). This physical contact is also used to guarantee the lactate/glucose dynamic in the CVU (Barros et al., 2007).

The contribution of vascular dysfunctions to Alzheimer's disease (AD) pathogenesis is now receiving increasing attention, especially in late-onset forms of the disease (Kapasi and Schneider, 2016). Recent imaging studies in preclinical and early AD have indicated that an impairment of the CVU leading to a reduction of the cerebral blood flow is an early event in AD (Garwood et al., 2016; van de Haar et al., 2016). However, the relative contribution of the different cell types and molecular mechanisms in CVU dysfunction, and its impact on neuron vulnerability is not clear. In fact, on one hand, neurons progressively accumulate amyloid peptide in the cytoplasm, leading to an increase of the intrinsic vulnerability (Baker-Nigh et al., 2015). On the other hand, astrocytes are subject to a number of cellular and molecular regulations related to the pathological microenvironment, including their activation as a consequence of AD neuroinflammation and amyloid plaques. It is not clear whether this results in neuroprotection, in further damage or in a biphasic effect, depending on the stage of the disease (Garwood et al., 2016).

*In vitro* models are useful to understand the relative contribution of intrinsic neuronal vulnerability due to β-amyloid (Aβ) peptide accumulation (Baldassarro et al., 2014) and astrocyte support associated with brain hypoperfusion. In particular, oxygen and glucose deprivation (OGD) is an *in vitro* model that mimics fundamental aspects of hypoperfusion (and ischemic) damage, i.e. low oxygen pressure and low nutrient levels (Goldberg and Choi, 1993). This model has been widely used to explore cellular and molecular mechanisms in experimental set-ups mimicking ischemic lesions and trauma (Cimarosti and Henley, 2008; Baldassarro et al., 2016). However, to the best of our knowledge, no studies have been published in which OGD is applied to *in vitro* cell systems appropriate for AD, i.e. which accumulate Aβ peptides (Baldassarro et al., 2014). Thus, the aim of the study was to establish a possible link between intraneuronal accumulation of Aβ and the vulnerability to a mild hypoxic/ischemic injury, using an *in vitro* model. First, we established an *in vitro* system of primary neurons and astrocytes derived from transgenic Tg2576 mice and wild-type controls. Tg2576 is a mouse model carrying a single human amyloid precursor protein mutation (APPswe) (Hsiao et al., 1996), and was chosen because of its predictive validity in pharmacological and non-pharmacological research targeting AD (Bilkei-Gorzo, 2014). These cell systems were then used to explore the contribution of intraneuronal Aβ accumulation and astrocyte-conditioned culture medium to neuron viability during OGD.

## RESULTS

### Cell system characterization and experimental design

**Fig. 1.**
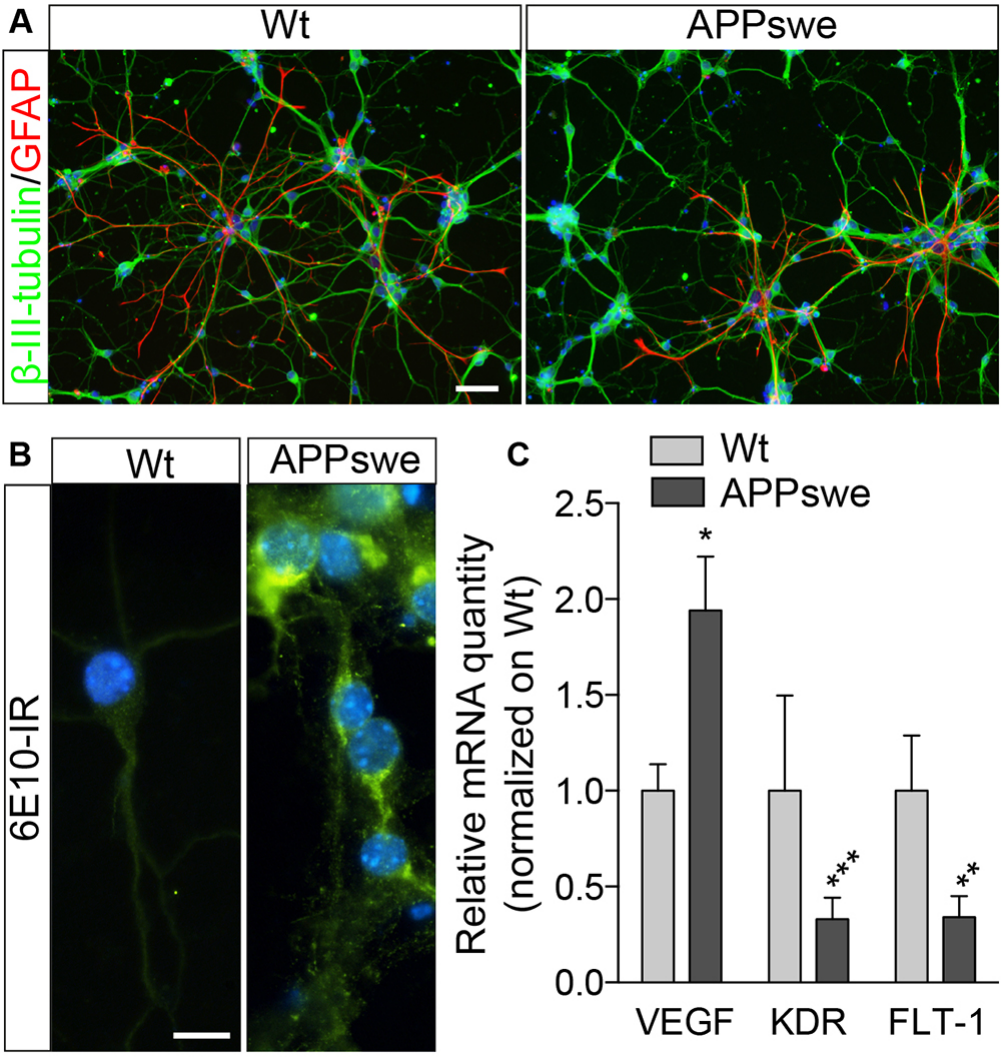
**Culture characterization.** (A) Representative images of double-stained cells showing β-III-tubulin-positive neurons and GFAP-positive astrocytes. Scale bar: 50 µm. (B) Representative images of 6E10-stained cells showing intracellular accumulation of the human APP protein/β-amyloid fragments. Scale bar: 10 µm. Nuclei are stained blue with Hoechst 33258. (C) mRNA expression level of VEGF and VEGF receptors FLT-1 and KDR (*VEGFA*, Wt *n*=15, APPswe *n*=11; *FLT1*, Wt *n*=13, APPswe *n*=11; *KDR*, Wt *n*=13, APPswe *n*=9). Bars represent mean+s.e.m. Statistical analysis: Student's *t*-test between genotypes (*P<0.05; **P<0.01; ***P<0.001).

Primary neurons were derived from the telencephalon of single pups, immediately characterized for the genotype. In this way, in each culture well, 100% of either wild-type (Wt) or APPswe neurons was seeded. Neurons were allowed to mature *in vitro* for 8 days, then characterized for cell composition by immunocytochemistry for neural (β-III-tubulin) and astroglial (GFAP) proteins (Fig. 1A). Both Wt and APPswe pure neuronal cultures contained a very low percentage of astrocytes (Wt, 3±2%; Tg2576, 2±3%), and no differences in cell composition between the genotypes were found. APPswe neurons were also characterized for amyloid peptide intracellular deposition using the 6E10 antibody. This antibody reacts with full-length amyloid precursor protein (APP) and the soluble form (sAPPα), as well as with the processed Aβ peptides. It is reactive to human-specific amino acid residues 1-6, within the amino acids 3-8 of Aβ. All neurons derived from Tg2576 mice show high intensity staining, whereas Wt neurons are negative (Fig. 1B). We also investigated the expression level of VEGFa and related receptors, a regulatory factor with a key role in angiogenesis, vascular development, and neuronal survival after ischemia (Takahashi and Shibuya, 2005). Notably, *VEGFA* mRNA expression level in APPswe is twice that of Wt (*P*=0.0274), while type 1 (*FLT1*) and type 2 (*KDR*) VEGF receptors are strongly down-regulated (FLT-1, *P*=0.0092; KDR, *P*=0.001; Fig. 1C).

### APPswe neurons are more vulnerable than Wt neurons

**Fig. 2.**
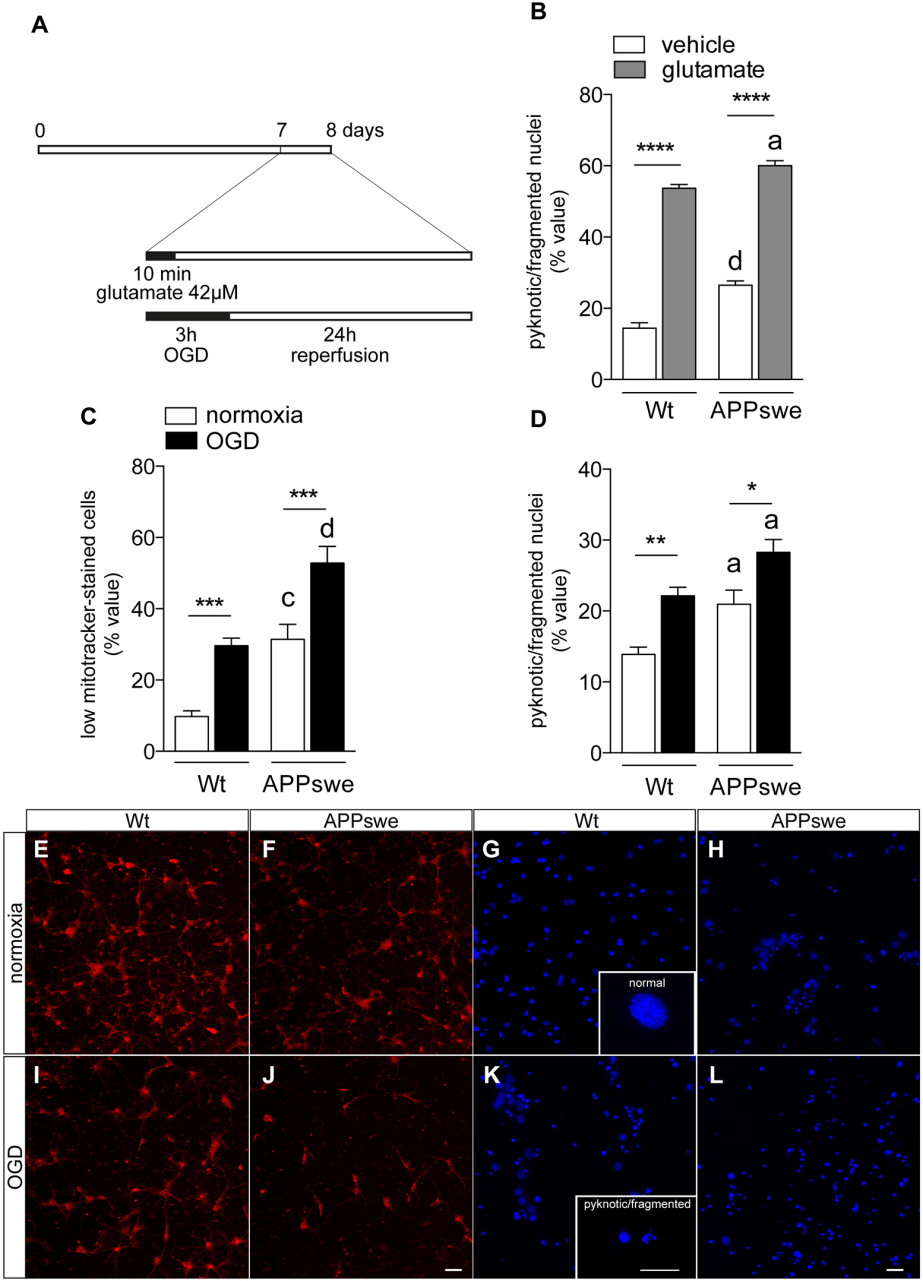
**Vulnerability of Wt and APPswe neurons to culture condition, glutamate excitotoxicity and OGD.** (A) Experimental design. Primary neurons isolated from Wt and Tg2576 mice were exposed at 7 DIV to the challenge stimulus (42 µM glutamate or 3 h OGD). Cells were then exposed to the original culture medium for 24 h. (B) Cell viability analysis of Wt and APPswe neurons exposed to vehicle and glutamate, as established by nuclear morphology (Wt vehicle, *n*=4; Wt glutamate, *n*=5; APPswe vehicle, *n*=5; APPswe glutamate, *n*=5). (C,D) Cell viability analysis of Wt and APPswe neurons exposed to normoxia and OGD, as established by mitochondrial function (C; Wt normoxia, *n*=10; Wt OGD, *n*=10; APPswe normoxia, *n*=8; APPswe OGD, *n*=9) and nuclear morphology (D; Wt normoxia, *n*=6; Wt OGD, *n*=5; APPswe normoxia, *n*=5; APPswe OGD, *n*=5). (E-L) Representative images of MitoTracker-stained cells (E,F,I,J) and Hoechst-stained nuclei (G,H,K,L) isolated from Wt (E,I,G,K) and APPswe (F,J,H,L) mice and exposed to normoxia (E-H) or OGD (I-L). Scale bar: 50 µm (E,F,I,J) and 80 µm (G,H,K,L). G and K include high-magnification images of a normal nucleus (G) or pyknotic/fragmented nuclei (K); scale bar: 10 µm. Bars represent mean±s.e.m. Statistical analysis: Two-way ANOVA, followed by Sidak's multiple comparison test. Asterisks represent differences between vehicle- and glutamate-treated groups (B; ****P<0.0001) or between normoxia- and OGD-exposed groups (C,D; *P<0.05; **P<0.01; ***P<0.001); letters represent differences between genotypes (a, *P*<0.05; c, *P*<0.001; d, *P*<0.0001).

We then challenged Wt and APPswe neurons under conventional experimental conditions to mimic *in vitro* hypoxic/ischemic brain conditions. In particular, glutamate excitotoxicity was established by 10 min exposure to 42 µM glutamate [EC_50_ at 7 days in vitro (DIV); Ha et al., 2009] followed by 24 h withdrawal; OGD was applied for 3 h, followed by 24 h reperfusion (Goldberg and Choi, 1993; Baldassarro et al., 2016) (Fig. 2A). Cell viability was established by the contemporaneous analysis of the mitochondrial membrane potential by MitoTracker and nuclear morphology by Hoechst 33258, using cell-based high-content screening as an analytical method. MitoTracker is a mitochondrial-selective fluorescent label that allows mitochondria depolarization, an early event in neurodegeneration, to be recognized in neurons (Lipton, 1999). OGD-induced cell death is characterized by mitochondria depolarization and cells showing depolarized mitochondria can be identified as poorly MitoTracker-labelled cells (Wappler et al., 2013; Wilson et al., 2014). Vulnerability of Wt and APPswe neurons to glutamate excitotoxicity is shown in Fig. 2B. APPswe neurons showed a higher cell death compared to Wt, both in the absence and in the presence of glutamate (Fig. 2D; treatment: *F*_1,15_, *P*<0.0001; genotype: *F*_1,15_, *P*<0.0001). OGD resulted in cell death, as evaluated by mitochondrial function (Fig. 2C; OGD: *F*_1,33_, *P*<0.0001; genotype: *F*_1,33_, *P*<0.0001) and nuclear fragmentation in both Wt and APPswe neurons (Fig. 2D; OGD: *F*_1,17_, *P*=0.0005; genotype: *F*_1,17_, *P*<0.0001). OGD produced stronger neuron degeneration in APPswe than in Wt neurons (mitochondria, *P*<0.0001; nuclei, *P*=0.0237). Representative images of MitoTracker-positive neurons in normoxia and under OGD are presented in Fig. 2E,F,I,J. Morphological criteria for automatically distinguishing normal versus pathological nuclei (pyknotic and fragmented) and representative images of Hoechst 33258-stained nuclei after normoxia or OGD exposure are presented in Fig. 2G,H,K,L.

### Intraneuronal amyloid increases neural vulnerability during OGD

**Fig. 3.**
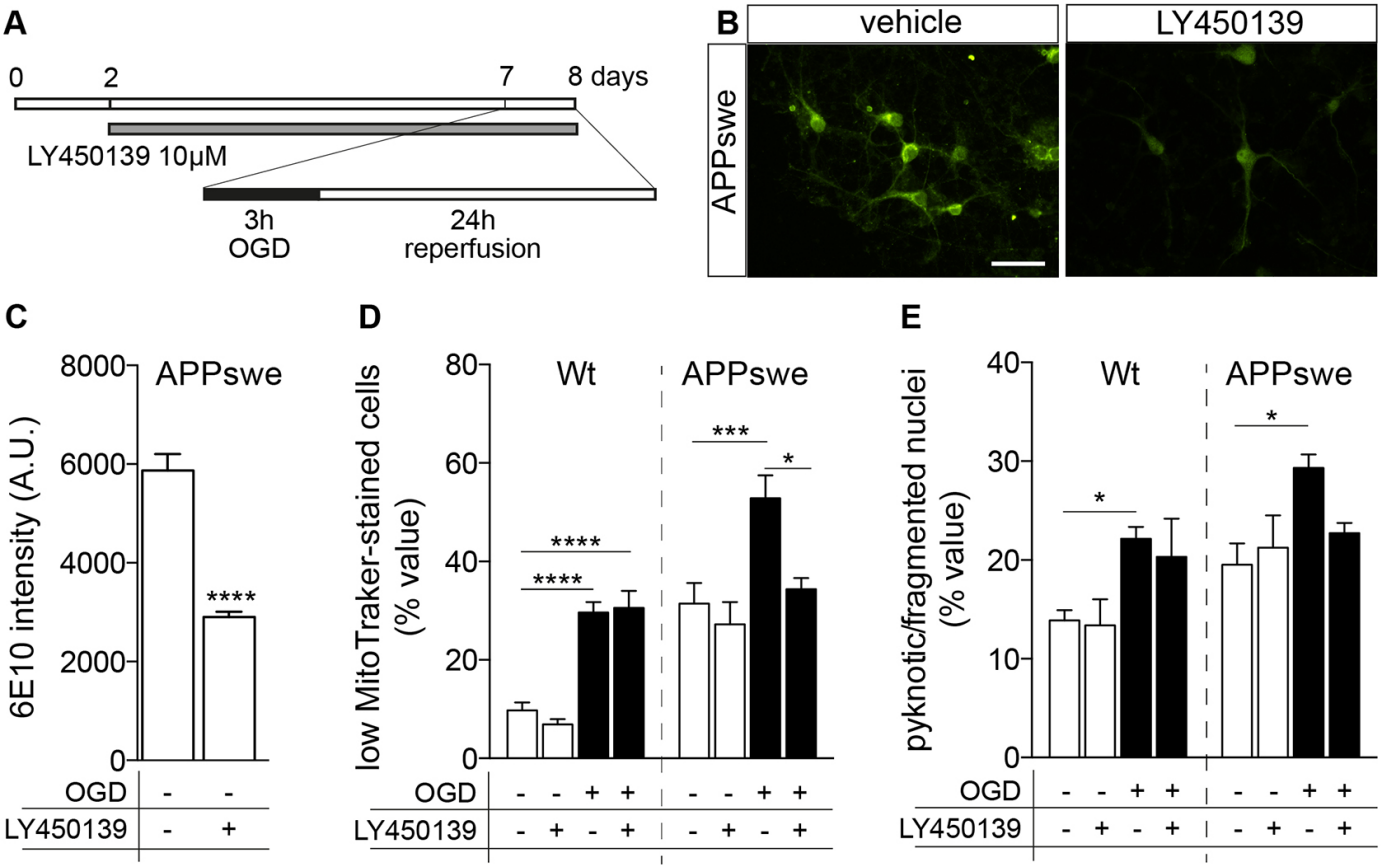
**Effect of γ-secretase inhibition on the vulnerability of primary neurons to OGD.** (A) Experimental design. Primary neurons isolated from Wt and Tg2576 mice were treated with LY450139 from 2 DIV to the end of the experiment. At 7 DIV, cells were exposed to 3 h of OGD and 24 h of reperfusion in the previous culture medium. (B) Representative images of 6E10 immunostaining of APPswe neurons, showing the intracellular accumulation of human APP protein/Aβ fragments. Scale bar: 50 µm. (C) Quantification of 6E10 in Tg2576 cells exposed to normoxia and treated or not with LY450139 (APPswe vehicle, *n*=5; APPswe LY450139, *n*=5). (D,E) Cell viability analysis of Wt and APPswe neurons exposed to normoxia and OGD, treated or not with LY450139, as established by mitochondrial function (E; Wt normoxia, *n*=10; Wt normoxia, LY450139, *n*=6; Wt OGD, *n*=10; Wt OGD LY450139, *n*=4; APPswe normoxia, *n*=7; APPswe normoxia LY450139, *n*=6; APPswe OGD, *n*=8; APPswe OGD LY450139, *n*=4) and nuclear morphology (F; Wt normoxia, *n*=6; Wt normoxia LY450139, *n*=4; Wt OGD, *n*=5; Wt OGD LY450139, *n*=4; APPswe normoxia, *n*=5; APPswe normoxia LY450139, *n*=4; APPswe OGD, *n*=5; APPswe OGD LY450139, *n*=5). Bars represent mean±s.e.m. Statistical analysis: one-way ANOVA followed by Tukey's multiple comparisons test inside the same genotype. Asterisks represent differences between LY450139- and vehicle-treated groups (*P<0.05; ***P<0.001; ****P<0.0001).

In order to establish if intraneuronal accumulation of Aβ peptides contributes to the increased vulnerability of APPswe neurons compared to Wt we used the γ-secretase inhibitor LY450139. This drug reduces both soluble Aβ and amyloid plaque burden in transgenic mice, lowering Aβ_40_ and Aβ_42_ production and secretion by the γ-secretase enzyme complex (Abramowski et al., 2008). Neuronal cultures were treated with the γ-secretase inhibitor LY450139, starting from 48 h after seeding and for the entire duration of the experiment (Fig. 3A). LY450139 (10 µM) inhibits the generation of Aβ peptides *in vivo* and *in vitro*, as also described for primary neurons transfected with APPswe (Elvang et al., 2009; Jämsä et al., 2011). This treatment also produces a substantial decrease of cytoplasmic 6E10-immunostaining in APPswe neurons (Fig. 3B,C), which proves the effectiveness of the γ-secretase inhibition, resulting in a reduction in the intracellular levels of APP/Aβ (Sivilia et al., 2013). Cells treated with LY450139 were then exposed to OGD and analysed for mitochondrial function (Fig. 3D) and nuclear morphology (Fig. 3E). LY450139 did not modify cell viability either in Wt or APPswe neurons, but it did abolish the mitochondria dysfunction induced by OGD in APPswe neurons (Fig. 3D; normoxia versus OGD, *P*=0.0028; OGD versus OGD+LY450139, *P*=0.0389) and afford partial protection against nuclear pyknosis (Fig. 3E; normoxia versus OGD, *P*=0.0268) .

### Astrocyte-conditioned medium after OGD protects APPswe neurons from OGD

**Fig. 4.**
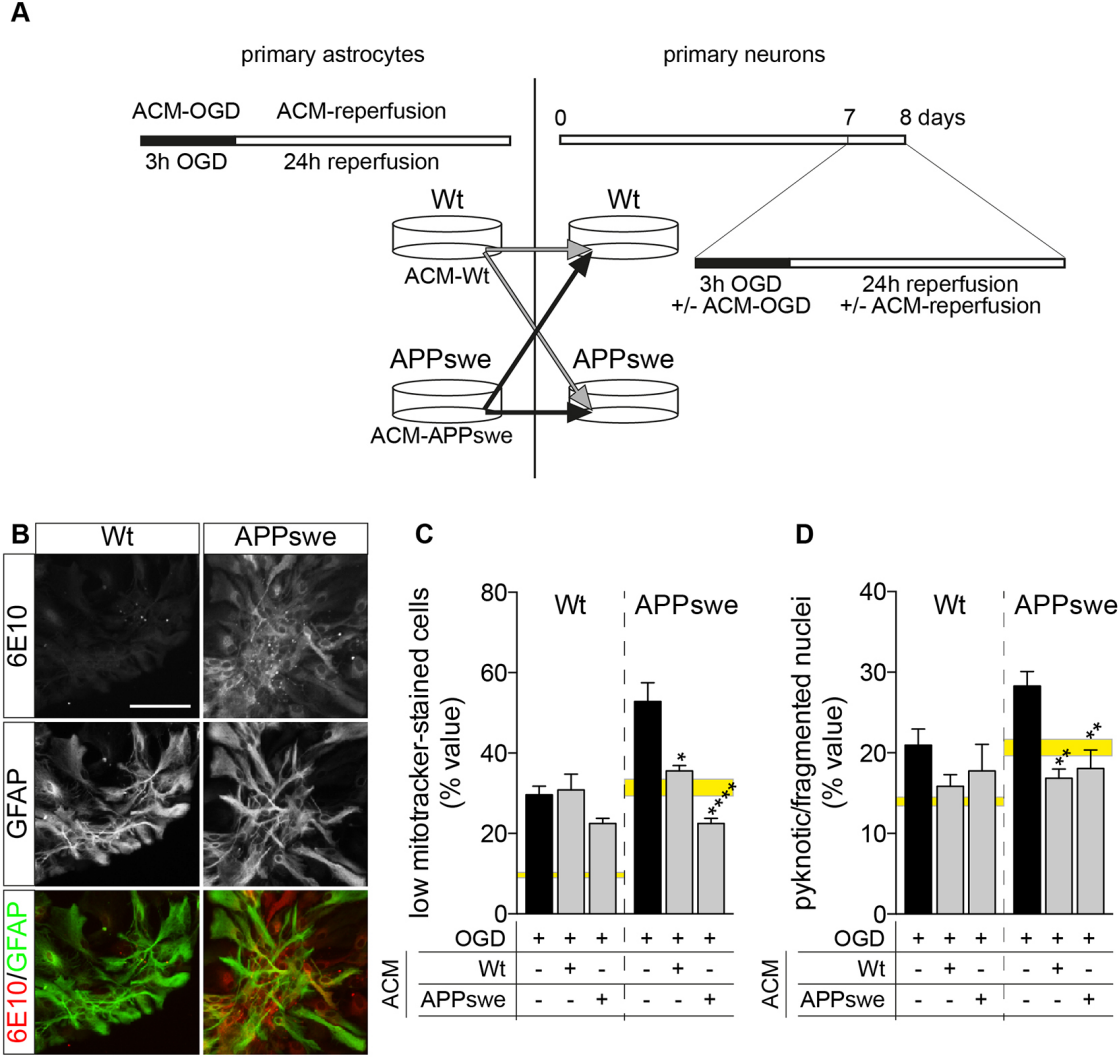
**Effect of astrocyte conditioned medium on the vulnerability of primary neurons to OGD.** (A) Experimental design. Primary astrocytes isolated from Wt and Tg2576 mice were exposed to 3 h OGD, and conditioned medium collected. Wt and APPswe primary neurons were then exposed to Wt and APPswe ACM during normoxia or OGD and reperfusion. (B) 6E10 immunostaining of APPswe astrocytes, showing the intracellular accumulation of human APP protein/Aβ fragments in GFAP-positive cells. Scale bar: 50 µm. (C,D) Cell viability analysis of Wt and APPswe neurons exposed to normoxia and OGD, treated or not with ACM, as established by mitochondrial function (C; Wt OGD, *n*=10; Wt OGD ACM-Wt, *n*=5; Wt OGD ACM-APPswe, *n*=6; APPswe OGD, *n*=9; APPswe OGD ACM-Wt, *n*=5; APPswe OGD ACM-APPswe, *n*=6) and nuclear morphology (D; Wt OGD, *n*=5; Wt OGD ACM-Wt, *n*=5; Wt OGD ACM-APPswe, *n*=4; APPswe OGD, *n*=5; APPswe OGD ACM-Wt, *n*=5; APPswe OGD ACM-APPswe, *n*=5). The yellow horizontal bars represent normoxia values (the height of the bar represent the range of the mean value±s.e.m.). Bars represent mean+s.e.m. Statistical analysis: one-way ANOVA followed by Dunnett's multiple comparison test inside the same genotype. Asterisks represent differences between ACM-treated groups and groups exposed to OGD only (*P<0.05; **P<0.01; ****P<0.0001).

In order to investigate the possible contribution of the paracrine properties of Wt and APPswe astrocytes in neuronal vulnerability after OGD, we prepared conditioned medium from astrocytes (ACM). The astrocytes were derived from Wt and APPswe mice. Cultures showing 98±2% of GFAP-positive cells and 6E10-immunoreactivity in APPswe GFAP-positive astrocytes are presented in Fig. 4B. We applied to astrocytes the same OGD protocol as used for neurons (3 h OGD and 24 h reperfusion). The culture medium was collected both after the OGD and reperfusion phases and used to treat primary neurons in the two phases of the experiment. Neuron viability after reperfusion was then established as mitochondria function (Fig. 4C) and nuclear morphology (Fig. 4D). ACM, whether from Wt or APPswe astrocytes, is not effective in protecting Wt and APPswe neurons from OGD. Conversely, ACM derived from both Wt and APPswe astrocytes fully protects APPswe neurons from OGD as measured by mitochondrial function (Fig. 4D; OGD versus ACM-Wt, *P*=0.113; OGD versus ACM-hAPP, *P*<0.0001) and number of pyknotic nuclei (Fig. 4D; OGD versus ACM-Wt, *P*=0.0011; OGD versus ACM-hAPP, *P*=0.0022). No statistical differences between Wt and APPswe ACM in protecting APPswe neurons were detected (Fig. 4C; one-way ANOVA followed by Tukey's *post hoc* Wt versus APPswe ACM, *P*=0.1039).

## DISCUSSION

Neurovascular abnormalities and aberrant glucose metabolism could be early events in the pathogenic cascade of AD and the underlying biological mechanisms are still obscure (Lourenço et al., 2015). In particular, there is a lack of studies aimed at examine the inter-related role of intracellular APP/Aβ peptide accumulation and neurovascular coupling during AD development and/or progression (Sagare et al., 2012). The aim of this study was to provide a novel *in vitro* system to dissect the relative contribution of neurons and astrocytes to cell vulnerability during hypoxic conditions in Alzheimer's disease. We thus derived primary cortical neurons and astrocytes from neonatal Tg2576 mice and Wt littermates. The experimental set-up included cells derived from single pups, which were split into different wells (technical replicates), with the single animal taken as the unit for statistical analysis (biological replicates). The experiments were analysed using a high-content approach, thus avoiding bias and providing a quite robust platform. Cell-based high-throughput technology combining cellular imaging with high-throughput data analysis (Radio and Mundy, 2008) has in fact been successfully applied to drug screening (O'Brien, 2014) and was also used to set up standard procedures according to the European Centre for the Validation of Alternative Methods (ECVAM) Good Cellular Culture Practice guidelines (Coecke et al., 2005). We first demonstrated that primary neurons derived from mice carrying the APPswe mutation, thus accumulating APP and amyloid peptides, are more vulnerable than neurons derived from Wt mice to conventional culture conditions, glutamate and OGD. We then demonstrated that the reduction in the concentration of amyloid peptides obtained by blocking γ-secretase enzyme synthesis results in a protection of APPswe neurons from OGD-induced degeneration. Finally, we showed that conditioned medium obtained from either Wt or APPswe astrocytes exposed to OGD is neuroprotective for APPswe, but not for Wt neurons.

The intracellular concentration of Aβ42, the most toxic Aβ variant, in pyramidal (CA1) human neurons has been estimated to be much higher in sporadic AD patients than in control subjects (Umeda et al., 2011). This is due to an early dysfunction of APP processing which occurs before extracellular plaque deposition (LaFerla et al., 2007; Gouras et al., 2010). Tg2576 mice also exhibit a high level of intraneuronal Aβ peptides at the pre-plaque stage (Balducci et al., 2011; Beggiato et al., 2014) and several working hypotheses have endeavoured to clarify the possible relationship between this event and cell vulnerability and death (Bramham, 2008; Crews and Masliah, 2010; Pensalfini et al., 2014). For example, intraneuronal Aβ perturbs local protein synthesis and cytoskeleton dynamics (Bramham, 2008) and is causally related to the activation of the protein kinases responsible for intracellular tau hyperphosphorylation and caspase-3 activation (Takahashi et al., 2002; Eimer and Vassar, 2013). In this study, we described how APPswe neurons are more vulnerable not only to OGD but also to glutamate excitotoxicity, that is the main driver for OGD-induced cell death (Goldberg and Choi, 1993), proving that the presence of the mutation influences the response of neurons to this stimulus. Treatment of the cultures with the γ-secretase inhibitor LY450139 results in neuroprotection, thus suggesting that intraneuronal Aβ accumulation could be responsible for the increased vulnerability to OGD observed in APPswe compared with Wt neurons. Actually, APPswe cells died in greater numbers in standard culture conditions, thus, the higher cell death under glutamate and OGD exposure could be an additive effect. However, the γ-secretase inhibition does not affect Wt viability nor the spontaneous cell death of APPswe neurons, strongly linking the inhibition of Aβ production to the protective effect.

In view of the key role of astrocytes in the CVU, we then performed experiments to dissect the possible interaction between astrocytes and neurons in our experimental systems, focusing on astrocyte-derived paracrine factors. Astrocytes are considered to be inherently neuroprotective in ischemic stroke and similar conditions (Zhao and Rempe, 2010), because of the role of astrocytes in supporting neuron energy homeostasis (Brown and Ransom, 2007). *In vitro*, co-cultures of neurons and astrocytes are more resistant to oxidative stress than pure neural cultures (Mattson and Rychlik, 1990). We have shown that conditioned medium obtained during OGD either from Wt or APPSwe astrocytes is also neuroprotective in APPSwe but not in Wt neurons. After 3 h OGD and 24 h reperfusion, the percentage of low-MitoTracker-stained cells in Wt is around 30% and in APPSwe is around 53%, while the percentage of pyknotic nuclei is 22% and 35%, respectively. After 3 h OGD and 24 h reperfusion, the percentage of low-MitoTracker-stained cells in Wt is 29.61±2.15% and in APPSwe is 54.92±4.76%, while the percentage of pyknotic nuclei is 22.16±1.18% and 28.28±1.81%, respectively. Thus, the OGD conditions used in this study can be considered ‘mild’ (Yu et al., 2012; Baldassarro et al., 2016). The ACM is not effective when used with Wt neurons, while it reduces the viability indices to Wt values when used with APPSwe neurons.

The reason for the selective effect of both ACM in APPSwe neurons only is not clear. In view of the role of astrocytes in CVU, the altered vascular endothelial growth factor (VEGFa) signalling observed in APPSwe- compared with Wt-derived cells could play a role. VEGFa and its receptors are in fact expressed in adult brain by neurons and astrocytes (Licht and Keshet, 2013; Mackenzie and Ruhrberg, 2012) and its expression is modulated by pathological conditions (Calzà et al., 2001) in humans (Boer et al., 2008) and in AD (Tarkowski et al., 2004). Here, we showed that APPSwe-derived neurons actually showed also a decreased expression of the relative receptors KDR and FLT-1. Moreover, the gene expression analysis of neurospheres derived from pre-plaque Tg2576 mice and analysed for clustering indicated that all upregulated genes in Tg2576 centred on VEGF (Baldassarro et al., 2013). It may thus be postulated that a different production of or sensitivity to paracrine factors occurs in APPSwe compared with Wt neurons. Several paracrine mechanisms between cells in the CVU playing a major role in neuron viability have been described *in vivo* and in *in vitro* systems, including growth factors (Lin et al., 2006; Li et al., 2014). Notably, VEGF seems to have a two-fold effect on neuron viability, which also depends on amyloid dosage (Sanchez et al., 2013; Dal Prà et al., 2014; Wood et al., 2015). Moreover, other potential cellular players for *in vivo* OGD neuroprotection, including endothelial cells and pericytes should be considered.

In conclusion, in this study we present an *in vitro* system aimed to dissect the contribution of the different cell types of the CVU to hypoxia and OGD. Primary neurons and astrocytes derived from transgenic animals carrying gene mutations of interest for AD are a promising tool for investigating co-morbidities and cofactors such as Aβ deposition and vascular dysfunction leading to hypoxia. Finally, the use of cell-based high-content analysis is recommended in order to improve *in vitro* data robustness.

## MATERIALS AND METHODS

### Primary neuronal cultures

All animal protocols described herein were carried out according to the European Community Council Directives (86/609/EEC), and comply with the guidelines published in the NIH Guide for the Care and Use of Laboratory Animals. Cortical neurons from single neonatal (within 24 h from birth) Wt or Tg2576 (Taconic, Hudson, NY, USA) mice were prepared according to standard protocols (Fernández et al., 2005; Del Vecchio et al., 2009). Briefly, brains were removed and cortical tissue dissected, freed from the meninges, and minced into small pieces. Cells were dispersed in Kreb's buffer (0.12 M NaCl, 4.8 mM KCl, 1.2 mM KH_2_PO_3_, 25.4 mM NaHCO_3_, 14.2 mM glucose, 0.01 mg/ml Phenol Red, 1.5 mM MgSO4) containing BSA 0.3% and 0.025% trypsin (Sigma-Aldrich) for 15 min at 37°C, followed by mechanical trituration with a Pasteur pipette in Kreb's buffer containing 0.004% deoxyribonuclease I (DNaseI, Sigma-Aldrich), and 0.052% soybean trypsin inhibitor (SBTI; Sigma-Aldrich). After centrifugation (500 g, 5 min), cells were resuspended in Neurobasal culture medium supplemented with 2% B27 (Invitrogen), 2 mM glutamine (Sigma-Aldrich), 100 U/ml penicillin, and 100 μg/ml streptomycin (pen/strep; Invitrogen) and plated onto Cultrex 2D substrate (0.25 mg/ml; Trevingen, Gaithersburg, MD, USA) coated plates or coverslips. Cells were maintained in a humidified incubator at 37°C with 5% CO2. To obtain neuronal culture (99% neurons) cells were treated after 24 h with 10 μM cytosine arabinofuranoside (Sigma-Aldrich) and at 4 DIV, half of the medium was changed. Neurons isolated from Tg2576 mice are denoted as APPswe primary neurons.

### Primary astrocyte cultures

Primary astrocytes were isolated from single 7-day-old Wt or Tg2576 mice using the same protocol as the primary neuronal cultures (Jones et al., 2011), except that cells were plated and maintained in DMEM with 15% fetal bovine serum (FBS), non-essential amino acid mixture (Sigma-Aldrich), pen/strep (Invitrogen) and 2 mM Glutamine (Invitrogen). Cultures were seeded in culture-treated flasks at a density of 125,000 cells/cm^2^ and maintained at 37°C 5% CO_2_. Cells were detached with trypsin (10 min, 37°C) and replated twice before use. For the OGD exposure, astrocytes were seeded in flat-bottom 96-well plates and exposed to OGD 2 weeks after plating. Astrocytes isolated from Tg2576 mice are denoted as APPswe primary astrocytes.

### Genotyping

Mouse tails were used for genotyping analysis. The mouse genomic DNA was extracted using the GenElute Mammalian Genomic DNA MiniPrep Kit (Sigma-Aldrich) according to the manufacturer's instructions and eluted in 100 µl of elution solution. DNA concentration was determined using a spectrophotometer and Tg2576 mice were identified by the presence of the mutated human APP gene (FW: 5′-GATGAGGATGGTGATGAGGTA-3′ REV: 5′-ACTGGCTGCTGTTGTAGG-3′) using the Real Time PCR technique and the SYBR Green qPCR master mix (Bio-Rad) and 0.4 µM forward and reverse primers. The amount of DNA used for each sample was 10 ng and PCR amplification conditions were: 60°C for 30 s.

### Glutamate excitotoxicity

At 7 DIV, Wt and APPswe primary neurons were treated with 42 µM glutamate. Briefly, medium was removed and cells were exposed to Krebs buffer, with or without glutamate, for 10 min. After glutamate treatment, Krebs buffer was replaced with medium (Fernandez et al., 2005).

### Oxygen and glucose deprivation and treatment

OGD was performed on primary cortical neurons and primary astrocytes cultures using an air-tight hypoxia chamber (Billups-Rothenberg, Del Mar, CA, USA) saturated with 95% N_2_, 5% CO_2_ (Goldberg and Choi, 1993). Glucose deprivation was achieved using a glucose-free Neurobasal medium, supplemented with B27, glutamine and penicillin/streptomycin as above. Oxygen was removed by flushing the hypoxia chamber with N_2_-CO_2_ mixture for 6-8 min at 25 l/min. The flushing was repeated after half the incubation time. The OGD condition was maintained for 3 h, after which plates were re-oxygenated for 24 h in the old medium in a cell incubator.

Wt and APPswe astrocyte media, both from the OGD and after reperfusion phases were collected, and used as astrocyte-conditioned medium (ACM) on neurons. In one set of experiments, Wt and APPswe primary cortical neurons were exposed to Wt and APPswe ACM both in the OGD and the reperfusion phases. In another set of experiments, primary cortical neurons were treated from 2 DIV to the end of the experiment (8 DIV) with 10 µM LY450139.

### MitoTracker staining

Cells were stained with MitoTracker Orange (Thermo Scientific, Waltham, MA, USA) following the manufacturer's instructions. Briefly, cells were treated for 30 min at 37°C with 150 nM MitoTracker. After two washes with PBS, cells were fixed and used in the immunocytochemistry procedure.

### Immunocytochemistry

At 8 DIV, cells were washed with ice-cold PBS and fixed in a solution of 4% paraformaldehyde for 20 min at room temperature (RT) and washed twice with PBS. Cells were then treated with 0.1 M PBS, 0.3% Triton X-100 containing 1% BSA and 1% normal blocking serum prepared from the species in which the secondary antibody was raised for 1 h at RT.

The following primary anti-sera were used: anti-6E10 (mouse; Covance, SIG-39320, batch no. D11AF00145; 1:1000), anti-β-III-tubulin (mouse; R&D Systems, MAB-1195, batch. no. HGQ0113121; 1:1000), anti-GFAP (rabbit; Dako, Z0334, batch no. 20005461; 1:1000) overnight at 4°C. Cells were washed with PBS and incubated with goat Alexa 488-conjugated anti-mouse, goat Alexa 568-conjugated anti-rabbit, goat Alexa 658-conjugated anti-mouse (Invitrogen) secondary antibodies for 30 min at 37°C. Cells were washed twice in PBS and incubated with the nuclear dye Hoechst 33258 (1 µg/ml) for 20 min at RT, washed with PBS and mounted with 0.1% para-phenylendiamine solution.

### Cell-based high-content screening

For HCS analysis cells were grown in 96 flat-bottom well HCS plates (Nunc, Roskilde, Denmark). Analysis of condensed nuclei, cell number and lineage/differentiation markers were performed with Cell Insight CX5 High Content Screening (HCS; Thermo Scientific), using the Compartmental Analysis BioApplication. Based on nuclear staining, the software is able to recognise nuclei and calculate the percentage of high intensity/small sized condensed nuclei. Moreover, based on nuclei identification, the software is able to detect the presence of the marker-specific stain in the cell body, calculating the percentage of immunoreactive cells and fluorescence intensity. For the MitoTracker analysis, the software is able to recognize every single cell by its nuclear staining and quantifies the fluorescence intensity inside the cell body. The operator can select a fluorescence threshold that allows the software to discriminate between ‘high-intensity staining’ and ‘low-intensity staining’. Using the same parameters and the same threshold it is possible to perform an automatic, statistically robust and objective analysis. This technique avoids also the bias of the analysis of randomly chosen fields, because the analysis is performed in all cells. From 60,000 to 80,000 cells/well were analysed.

### RNA isolation and reverse transcription

Total RNA isolation was performed with the RNeasy Micro kit (Qiagen) following the manufacturer's instructions. Total RNA was eluted in RNase-free water and concentration estimated through absorbance values at 260, 280 and 320 (Nanodrop 2000 spectrophotometer, Thermo Scientific). First-strand cDNAs were obtained using the iScript cDNA Synthesis Kit (Bio-Rad), incubating at 42°C for 30 min. An RNA sample with no reverse transcriptase enzyme in the reaction mix was processed as a no-reverse transcription control sample.

### Semi-quantitative real-time PCR

Semi-quantitative real-time PCR was performed using the CFX96 real-time PCR system (Bio-Rad). The reactions were performed in a final volume of 20 µl consisting of 1× SYBR Green qPCR master mix (Bio-Rad) and 0.4 µM forward and reverse primers. In order to avoid possible contamination of genomic DNA in isolated RNA, the sample with no reverse transcriptase enzyme was processed in parallel with the others and tested by real-time PCR for every pair of primers used. All primers used were designed using Primer Blast software (NCBI) and synthesized by IDT (Coralville). The following primer sequences were used: *VEGFA* (FW: 5′-AAGAGAAGGAAGAGGAGAG-3′; REV: 5′-ACCCAAGAGAGCAGAAAG-3′); *FLT1* (FW: 5′-CGTGCAAGGAACCTCAGACA-3′; 5′-ATCATAGGGCAGCCGTTCAC-3′); *KDR* (FW: 5′-ATGTCCTTGGCTGTGCAAGA-3′; REV: 3′-CCTTCATTGGCCCGCTTAAC); *GAPDH* (FW: 5′-GGCAAGTTCAATGGCACAGTCAAG-3′; REV: 5′-ACATACTCAGCACCAGCATCACC-3′) was used as a housekeeping gene to normalize the amount of reverse-transcribed RNA used for PCR. Thermal profile of PCR reactions consisted first of a denaturation step (95°C, 2 min) and 40 cycles of amplification (95°C for 15 s and 60°C for 60 s). At the end of the amplification cycles, the melting curve of amplified products was performed according to the following temperature/time scheme: heating from 55°C to 95°C with a temperature increase of 0.5°C/s.

Primer efficiency values for all primers were 95-102%. The 2^(−ΔΔCT)^ method was used for the calculation of gene expression.

### Statistical analysis

The number of wells per animal varied according to the cell isolation yield. For mRNA analysis, each value derived from a single animal. For HCS analysis, individual data were obtained from the mean value of the wells obtained from a single animal culture. Exclusion criteria in data analysis were pre-established for each assay as follows. mRNA analysis: samples in which curves not fitting the melting temperature or in which a double peak was observed were excluded from the analysis; HCS analysis: wells in which the software was unable to automatically identify the nuclei were excluded from the analysis. The final number of data per group is reported in the figure legends.

Data are reported as mean±s.e.m. Prism software (GraphPad) was used for statistical analyses and graph generation. Student's *t*-test, two-way ANOVA and Sidak's multiple comparison or one-way ANOVA and Dunnett's or Tukey's multiple comparison *post hoc* were used to analyse data, as specified in the figure legends. Results were considered significant when the probability of their occurrence as a result of chance alone was less than 5% (*P*<0.05).
